# ChatGPT and BCI-VR: a new integrated diagnostic and therapeutic perspective for the accurate diagnosis and personalized treatment of mild cognitive impairment

**DOI:** 10.3389/fnhum.2024.1426055

**Published:** 2024-06-04

**Authors:** Yiduo Yao, W. Z. W. Hasan, Wenlong Jiao, Xianling Dong, H. R. Ramli, N. M. H. Norsahperi, Dong Wen

**Affiliations:** ^1^Department of Electrical and Electronic Engineering, Faculty of Engineering, University Putra Malaysia, Selangor, Malaysia; ^2^Brain Computer Intelligence and Intelligent Health Institute, School of Intelligence Science and Technology, University of Science and Technology Beijing, Chengde, China; ^3^Hebei International Research Center of Medical Engineering, Chengde Medical University, Hebei, China

**Keywords:** brain-computer interface, virtual reality, mild cognitive impairment, ChatGPT, diagnostic, therapeutic

## 1 Introduction

The large language model, ChatGPT (OpenAI, 2024), launched by OpenAI on November 30, 2022, rapidly propelled it into the world's fastest-growing consumer software applications, attracting over a hundred million users. This milestone not only marks a significant achievement in artificial intelligence (AI) but also has sparked widespread global interest in the potential applications of AI, particularly in the medical domain (Biswas, 2023; Wójcik et al., 2023). Especially in areas such as diagnostic assistance, treatment planning and recommendations, clinical practice and guidelines, clinical research and data analysis, telemedicine, and medical resource allocation (Ferdush et al., 2023), ChatGPT has shown immense potential.

The accurate diagnosis and effective treatment of Mild Cognitive Impairment (MCI) are fraught with numerous challenges, including the high costs and invasive nature of traditional diagnostic methods, side effects associated with treatment plans, and the limitations of resources and time for non-pharmacological interventions (Aniwattanapong, 2021; Reynolds et al., 2021). Given these challenges, improving the accuracy and efficiency of MCI diagnoses and optimizing patient treatment plans have emerged as crucial issues needing resolution in this field.

Simultaneously, the rapid advancement of Brain-Computer Interface (BCI) and Virtual Reality (VR) technologies offers new avenues for addressing these issues. These technologies not only make the diagnostic and treatment processes more convenient and intelligent but also enable BCI-VR-based diagnostic and therapeutic technologies to provide more rapid and accessible methods of diagnosis and assessment for patients with cognitive impairments (Lee et al., 2013).

This paper explores how BCI-VR technologies and ChatGPT can be integrated within medical applications, aiming to propose a comprehensive diagnostic and treatment framework specifically designed for patients with cognitive impairments. By integrating BCI, VR, and ChatGPT, this platform not only seeks to provide patients with new pathways for remote diagnostics, treatment, and follow-up visits but also aims to establish a scientific and systematic framework for comprehensively evaluating the benefits, potential risks, and challenges inherent in technological integration, paving the way for future advances in the diagnosis and treatment of cognitive impairments.

## 2 Basic overview of the diagnosis and therapeutic of MCI

MCI typically involves early-stage changes in cognition that precede more severe neurodegenerative disorders. These changes are often characterized by synaptic dysfunction and abnormal protein accumulations, such as amyloid-beta plaques and tau protein tangles, which affect memory and cognitive functions (Tremblay et al., 2007; Canuet et al., 2015; Gonzales et al., 2018; Yin et al., 2021; Contador et al., 2023; Carvalho et al., 2024).

MCI serves as a critical transitional stage between normal aging and dementia. Diagnosis involves clinical observation (Rodriguez-Santiago et al., 2024), neuroimaging (Chouliaras and O'Brien, 2023), biomarkers (An et al., 2024; Carpi et al., 2024; Kim S. et al., 2024; Kim S. K. et al., 2024; Perneczky et al., 2024; Plassman et al., 2024; Wu et al., 2024; Zuo et al., 2024), and electroencephalography (EEG) (Amato et al., 2024; Faghfouri et al., 2024; Khatun et al., 2024; Rutkowski et al., 2024; Sun et al., 2024), as well as neuropsychological assessments.

However, each diagnostic method has its limitations. Cognitive scales, while practical, are vulnerable to subjective inaccuracies (Chouliaras and O'Brien, 2023; Tsushima et al., 2024). Biomarkers, although precise, are invasive and expensive (An et al., 2024; Carpi et al., 2024). EEG can reveal subtle brain function changes but requires specialized expertise for analysis (Meghdadi et al., 2021). These challenges necessitate comprehensive data analysis, which is often laborious.

MCI treatment includes pharmacological and non-pharmacological interventions. Pharmacological methods can alleviate symptoms but are costly and carry adverse side effects (Kho et al., 2021; Nguyen et al., 2021; dos Santos Moreira et al., 2022; Zhang et al., 2022; Yu et al., 2023). Non-pharmacological treatments like cognitive training and lifestyle changes face resource and time limitations (Aniwattanapong, 2021; Reynolds et al., 2021).

Remote cognitive training and rehabilitation offer convenience but often lack sufficient rigor and structure (Niama Natta et al., 2015; Gately et al., 2019). As a result, improved diagnostic and treatment solutions that are both precise and individualized are urgently needed.

## 3 Current applications status of BCI-VR in MCI diagnosis and treatment

The combination of BCI and VR technologies has sparked a wave of technological innovation in the medical field, particularly demonstrating the immense value of their application in diagnosing and treating cognitive impairments. BCI technology interprets brain activity to generate control signals, while VR technology provides an immersive experience, showing practical potential in diagnosing and treating patients with MCI.

Research on diagnostic and therapeutic approaches for spatial orientation impairments has shown that targeted VR games integrated with BCI technology can assess and enhance patients' spatial cognition. For instance, simulations of supermarkets (Zygouris et al., 2017), hospital environments (Jiang and Li, 2007), and road navigation systems (Liu et al., 2022) provide real-time neural feedback, adapting interventions to individual needs.

In virtual life scenes and simulated driving tasks, BCI allows precise evaluation and targeted enhancement of episodic memory (Bellassen et al., 2012; Sauzéon et al., 2016). Similarly, with BCI data, VR simulations of daily environments like buses and kitchens facilitate personalized cognitive rehabilitation (Richard et al., 2010; Yamaguchi et al., 2012; Allain et al., 2014; Vallejo et al., 2017).

For motor and balance impairments, BCI-VR tasks, such as simulated cycling, provide neural monitoring for tailored training, revealing their value in improving executive functions and memory (Arciero et al., 2020).

Integrating BCI and VR technologies allows for a direct connection to the patient's nervous system and the creation of immersive therapeutic environments. However, the BCI-VR system cannot directly analyze the patient's condition nor adjust the treatment plan in real-time. By incorporating large language models into the diagnosis and treatment of MCI, it would be possible to enhance the accuracy of MCI diagnoses further and provide patients with more direct and personalized diagnostic and therapeutic methods.

## 4 Current application status of ChatGPT in MCI diagnosis and treatment

Integrating large-scale language models into BCI applications represents a significant advancement in neurotechnology. These models possess sophisticated computational abilities that can improve the interpretation and utilization of EEG signals. Accurate decoding and transformation of EEG data are crucial for effective brain-device communication, and large-scale language models, with their advanced pattern recognition and machine learning algorithms, are uniquely positioned to enhance this process. Learning from vast neural datasets enables the development of highly accurate and responsive BCI systems, which are essential for assistive technologies and advanced neurofeedback systems.

In the field of BCI applications, large-scale language models such as GPT (Cui et al., 2023; Kim J. W. et al., 2024), BERT (Devlin et al., 2018; Kostas et al., 2021), and Seq2Seq (Sutskever et al., 2024) have demonstrated their unique characteristics and applicability. The GPT series is renowned for generating coherent and contextually relevant text outputs, showcasing a deep understanding of complex contexts. However, its direct application in decoding electroencephalography signals is limited, requiring reliance on external conversion systems (Cui et al., 2023; Kim J. W. et al., 2024). In contrast, while the BERT series excels in text comprehension, it has comparatively weaker capabilities in generating text for specific BCI tasks, necessitating model fine-tuning for optimization (Ethayarajh, 2019; Rogers et al., 2021). Seq2Seq models can directly transform EEG signals into text sequences, supporting the realization of more natural language dialogue systems but face challenges in managing long-distance dependencies and high training complexity (Luong et al., 2015; Keneshloo et al., 2019; Shi et al., 2021; Wang et al., 2022).

Considering these models' performance, advantages, and disadvantages, this study has chosen to investigate the ChatGPT model further, finding it to exhibit significant potential in the medical field. The application of ChatGPT across various medical subdomains, including neurodegenerative diseases (Koga et al., 2023), neurosurgical procedures (Liu et al., 2023), psychiatric diagnoses, and oncology (Haemmerli et al., 2023; Li et al., 2023; Schulte, 2023; Gosak et al., 2024; Yang et al., 2024), has proven effective in assisting diagnoses and offering treatment suggestions (Au and Yang, 2023; Delsoz et al., 2023; Haemmerli et al., 2023; Yeo et al., 2023). This model, integrated with deep learning techniques, can analyze EEG or MRI data (Boßelmann et al., 2023), identifying subtle changes, thus assisting physicians in conducting comprehensive risk assessments and developing personalized treatment plans (Abani et al., 2023; Zheng et al., 2024). Furthermore, through interaction with patients, ChatGPT can monitor changes in cognitive status and provide personalized cognitive training and psychological support, offering substantial support for treating cognitive disorders such as dementia (Zheng et al., 2024). Notably, ChatGPT, with its efficient capabilities in parsing and generating human language, has shown proficiency in accurately interpreting medical literature and engaging in empathetic patient communication (Dave et al., 2023; Ferdush et al., 2023) significantly boosting the auxiliary diagnostic and therapeutic potential for MCI.

The use of ChatGPT in diagnosing and treating MCI signifies a significant leap in AI within healthcare, improving diagnostic precision, treatment approaches, and patient interaction (Wójcik et al., 2023). ChatGPT's success points to the vast potential for advanced language models in medicine, especially alongside technologies like BCI and VR. This interdisciplinary approach innovates MCI management and paves the way for efficient, personalized healthcare solutions.

## 5 Our viewpoint: new perspective on the fusion of ChatGPT and BCI-VR systems in the diagnosis and treatment of MCI

ChatGPT, as a sophisticated language model, is adept at monitoring nuanced changes in linguistic patterns that indicate cognitive decline, such as reduced lexical diversity, simplified syntax, increased speech latency, and disjointed semantic coherence. It interacts with patients by acting as a conversational agent to engage in dialogue, analyze symptom reports, and guide them through cognitive tasks, which are crucial for assessing cognitive functions like memory and executive skills. This ongoing assessment is less invasive and can be conducted in real time, allowing ChatGPT to establish a linguistic baseline and monitor deviations over time. The collected data are analyzed using advanced machine learning techniques to detect trends that may predict cognitive impairment progression, offering a valuable tool for early diagnosis and continuous monitoring in cognitive healthcare.

Integrating ChatGPT into a cognitive disorder diagnosis and treatment system based on BCI and VR technologies showcases a new perspective on technological innovation. This system offers a multifaceted integrated solution by combining the immersive experience of VR, the precise brain activity-capturing capabilities of BCI, and ChatGPT's advanced natural language processing (NLP) functions. Such technological integration not only innovates in treatment methodologies but also represents a qualitative leap at the technological level, bringing cognitive training and rehabilitation activities closer to complex real-world scenarios. Moreover, the application potential of this system spans patient diagnostic assistance, personalized treatment plan formulation, and data analysis for doctors (Ferdush et al., 2023), providing comprehensive support and fresh perspectives for the diagnosis and treatment of cognitive disorders.

**Figure 1:**
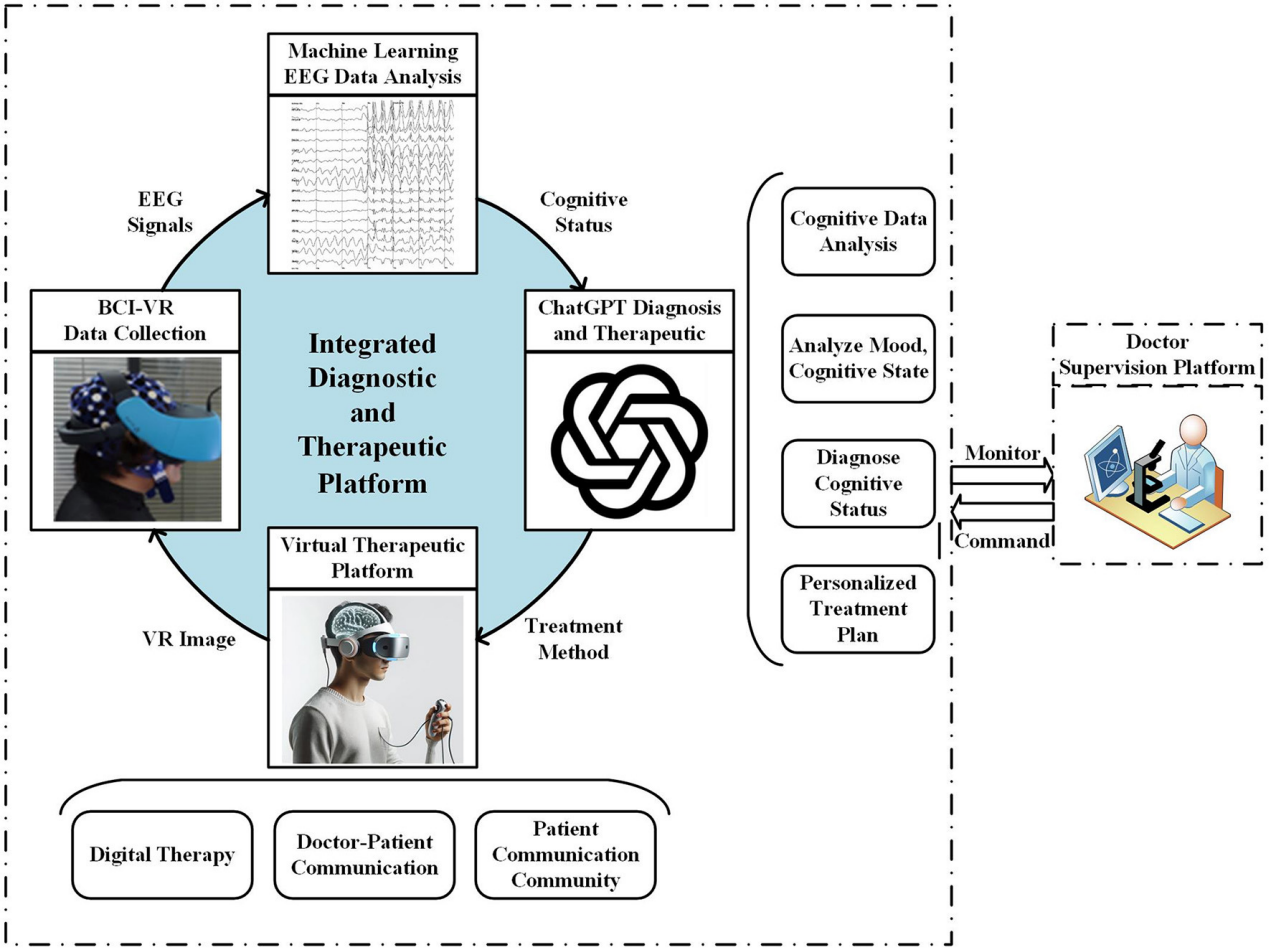
MCI diagnosis and therapeutic platform based on ChatGPT and BCI-VR.

As depicted in Figure 1 (Visio, 2021), the system harnesses BCI and VR technologies to immerse patients in realistic virtual environments where cognitive testing is conducted. Patients don a BCI-VR helmet that facilitates the real-time capture and analysis of neural signals associated with specific cognitive functions. This integration allows for precise monitoring of brain activity during VR-based cognitive exercises.

The system utilizes ChatGPT's advanced NLP capabilities to dynamically interpret patients' verbal and behavioral responses. This interaction augments diagnostic accuracy and enhances patient engagement by providing immediate and personalized feedback. For instance, ChatGPT can elucidate test results on the fly and offer tailored medical advice based on the patient's performance and responses during the VR activities.

In addition to diagnostic applications, the platform incorporates therapeutic tools, such as games and music, designed to bolster cognitive function and emotional wellbeing. These tools are accessible within the VR environment, enabling social interactions through multiplayer setups that allow patients to connect with family, friends, or other patients, thereby supporting their social and emotional health.

Furthermore, the platform integrates a telemedicine feature that facilitates direct communication between patients and healthcare providers. This feature supports real-time, face-to-face interactions within the virtual space, enabling physicians to make informed decisions about treatment strategies based on comprehensive data analysis. The system's capacity to merge detailed EEG data analysis with behavioral observations ensures that treatment plans are precise and personalized, reflecting the system's overarching aim to enhance cognitive impairment therapies' accuracy and efficacy.

## 6 Risks and challenges

The integration of VR and BCI technologies, enhanced by large-scale language models like ChatGPT, epitomizes the forefront of technological innovation in cognitive diagnostics and therapy. While these technologies offer substantial benefits in medical rehabilitation, entertainment, and assistive devices for disabled people and promise to improve the quality of life and rehabilitation outcomes for cognitively impaired patients, their full potential is tempered by significant challenges.

Seamless technical integration demands interdisciplinary collaboration to ensure compatibility across diverse operating systems and hardware platforms (Putze et al., 2020). User acceptance is complicated by adaptation barriers and trust issues, particularly among the elderly, who may also experience discomfort from VR and reservations about BCI technologies.

Data privacy and security are paramount, given the sensitivity of EEG signals and behavioral data captured in VR environments. Robust encryption and stringent security protocols are essential to safeguard patient data (Li et al., 2015). Furthermore, maintaining accuracy and reliability in long-term applications poses challenges that impact the stability and effectiveness of these diagnostic and therapeutic systems.

Regulatory and ethical considerations are crucial, requiring careful management of informed consent, privacy rights, and user autonomy while minimizing the risk of BCI technology misuse (Burwell et al., 2017; Coin, 2020). Addressing these issues necessitates a concerted effort involving technological innovation, user education, and rigorous clinical research, ensuring that the potential of BCI-VR systems in cognitive health care is realized responsibly and effectively.

## 7 Conclusion

Through a comprehensive literature analysis, this study has uncovered the potential application value of ChatGPT in treating cognitive disorders, mainly through its integration with BCI and VR systems, proposing innovative solutions for diagnosing and treating cognitive disorders. Initially, the study assessed the limitations of traditional diagnostic and treatment methods for cognitive disorders, followed by a detailed analysis of the application of BCI-VR technology and ChatGPT in this field. It explored the feasibility and potential trends of integrating ChatGPT with BCI-VR systems in practical applications. Despite some challenges and gaps in the real-world application of this technological integration, this research suggests that BCI-VR systems incorporating large language models like ChatGPT have broad application prospects.

## Author contributions

YY: Writing – original draft, Writing – review & editing. WH: Supervision, Writing – review & editing. WJ: Methodology, Writing – review & editing. XD: Writing – review & editing. HR: Writing – review & editing. NN: Writing – review & editing. DW: Supervision, Writing – review & editing, Funding acquisition.
